# Decarbonising existing homes in Wales: a participatory behavioural systems mapping approach

**DOI:** 10.14324/111.444/ucloe.000047

**Published:** 2022-11-09

**Authors:** Joanna Hale, Christopher Jofeh, Paul Chadwick

**Affiliations:** 1 Centre for Behaviour Change, University College London, London, UK; 2 Welsh Government’s Independent Advisory Group on Residential Decarbonisation

**Keywords:** energy, decarbonisation, retrofit, behaviour change, complexity, systems thinking, participatory systems mapping, policy

## Abstract

To reduce carbon emissions, urgent change is needed to high-carbon human behaviours including home energy use. Previous policy failures point to insufficient integration of systemic and behavioural approaches which are too often seen as alternative and incompatible approaches to bring about change. A novel behavioural systems mapping approach was used to inform national policy recommendations for energy-saving retrofit of homes in Wales. Three participatory workshops were held with the independent Welsh residential decarbonisation advisory group (‘the Advisory Group’) to: (1) map relationships between actors, behaviours and influences on behaviour within the home retrofit system; (2) provide training in the Behaviour Change Wheel (BCW) framework and (3) use these to develop policy recommendations for interventions. Recommendations were analysed using the capability, opportunity and motivation (COM-B) model of behaviour to assess whether they addressed these factors. Two behavioural systems maps (BSMs) were produced, representing privately rented and owner-occupied housing tenures. The main causal pathways and feedback loops in each map are described. Necessary interventions to achieve national-scale retrofit included: government-led investment, campaigns and awareness-building, financial-sector funding mechanisms, enforcement of regulations and creating more streamlined and trusted supply chain services. Of 27 final policy recommendations, six addressed capability, 24 opportunity and 12 motivation. Participatory behavioural systems mapping can be used in conjunction with behaviour change frameworks to develop policy recommendations that address the behavioural determinants of complex environmental problems in a systemic way. Research is underway to refine and extend the approach through application to other sustainability challenges and methods of constructing systems maps.

## Introduction

The climate crisis requires governments to bring about behaviour change across populations at a scale and rapidity that has never been seen before. In the UK, climate policy advisers have grouped actions needed to reach net zero emissions according to whether they are expected to be delivered through behaviour change, technology change or a combination [1,2]. However, all actions to reduce carbon emissions, including technology changes, are embedded in complex systems of behaviours performed by multiple actors at local, regional, national and international scales [1,3–6]. To develop effective climate policies, governments and other decision-makers are therefore encouraged to use interdisciplinary, systemic and participatory approaches to integrate expertise from multiple sectors and stakeholder groups [3,7–11].

A range of approaches under the umbrella of participatory systems mapping have been applied to environmental policy problems [7]. However, few participatory systems mapping methods focus explicitly on the behaviours of different actors, and none are directly linked to or informed by behaviour change frameworks. Until recently, ‘systems change’ and ‘behaviour change’ have often been positioned as alternative policy approaches, with an implied tension or trade-off between interventions that target technological, infrastructural and regulatory systems versus interventions that target individual citizens’ knowledge or motivation [12]. Behavioural and social scientists have pointed out that this dichotomy is inaccurate and unhelpful [5,6]. Instead, there are calls for research to focus on ways that tools for analysing complex systems can be combined with theoretical models about individual and collective behaviour, so as to develop more successful interventions [13–15].

In this paper we describe a behavioural system mapping approach, linked to the BCW framework [16], used to combine expertise from multiple sectors and disciplines for the purpose of developing policy recommendations with population-level behaviour change as their primary objective. Specifically, we focus on the major challenge of retrofitting existing homes in Wales to reduce energy consumption.

## The challenge of retrofitting existing homes in Wales

To meet the UK’s target of net zero carbon emissions by 2050, 87% of the existing housing stock – around 25 million homes – must be made energy efficient [17–19]. In Wales, domestic energy use is responsible for approximately 27% of national energy consumption and the majority of this is used on heating [20]. The existing housing stock is one of the oldest in Western Europe with most properties having low levels of heat integrity, meaning that much of the energy used is wasted, exacerbating fuel poverty problems [21]. Owner-occupiers and private tenants occupy 85% of homes in Wales [22], and therefore understanding how people in these tenures can be supported to increase the energy efficiency of their homes is a high priority.

Existing homes will require retrofitting to make them energy efficient. The UK Committee on Climate Change (CCC) and the Construction Leadership Council (CLC) emphasise this as an urgent priority [23,24]. Retrofit refers to a range of activities to improve the energy efficiency of buildings through repairs, upgrades and maintenance to the building itself, as well as changes to power and heat provision and user controls [25,26]. Examples of retrofit measures include: thermostatic radiator controls, draft proofing, insulation, window replacement, photovoltaic cells (solar panels) and heat pumps [26]. The variation in the existing housing stock in Wales means that there is no one-size-fits-all solution, and a range of approaches tailored to individual buildings are needed [27], which may be time sensitive [28]. The scale of the technical challenge in the UK is such that the CLC estimates it will take an additional workforce of 500,000 and the CCC recommends immediate government action.

Retrofit has previously been seen as a technical and economic challenge [4], but this view has shifted in light of the failure of previous policies to achieve impact at scale. For example, whilst the Green Deal, introduced in 2013, used a market-led approach to stimulate the private sector to design and deliver retrofit options, it failed to attract private investors or households in large numbers [29–31]. Analyses of the failures of previous initiatives suggest the following factors need to be considered in future policy approaches: first, greater integration of expertise from multiple sectors and disciplines is needed [31,32]; second, the behaviours and motivations of a wide range of actors need to be better understood and addressed [4,25,33]; and third, there are likely to be unintended consequences of policies intended to improve energy efficiency of the housing stock [18,34], including the possibility of creating or widening social injustices [35,36].

These considerations suggest that home retrofit needs to be understood as part of a complex sociotechnical system, and greater attention paid to the diversity of actors involved [33,37–41]. Key groups of people in the home energy system include homeowners and national policymakers, as well as others that have been considered more ‘hard to reach’. In particular, people within the repair, maintenance and improvement (RMI) sector – a key part of the supply chain – are highly influential on the adoption of energy saving measures in homes, but have previously been outside the reach of policy debates and interventions around retrofit [25,26,42]. Others include people working within and for local authorities, previously described as marginal players in UK energy efficiency policy [30] despite their strategic positioning to help deliver retrofit at scale [43], as well as people and organisations within the financial sector who could provide novel models and products to finance home retrofitting [44].

Meeting the challenge of retrofitting owner-occupied and privately rented homes at scale in Wales will therefore require understanding how the behaviours of multiple actors within this complex system influence homeowners to *install* energy saving measures and *use* them properly. This means that knowledge exchange between different disciplines and sectors will be essential [32]. In line with this need, the independent Welsh residential decarbonisation advisory group (‘the Advisory Group’) was created in 2018 to make policy recommendations for a long-term programme of decarbonising housing. The Advisory Group established and brought together five subgroups to consider interconnections between technical and infrastructure, governance, community benefits, customer confidence and financial issues. The Advisory Group sought to synthesise knowledge across the five areas and combine this with an understanding of how to intervene and change the behaviours of actors within complex systems. In the next section, we introduce and describe the behavioural systems mapping approach which was developed to help achieve this aim.

## Participatory approaches to systems mapping

There are a wide variety of systems approaches used in academic and applied settings to assist policy development and decision-making. Examples include, but are not limited to: journey mapping, network analysis, agent-based modelling, fuzzy cognitive mapping, rich pictures, causal loop diagrams (CLDs) and stock-and-flow modelling. Although these approaches have roots in different fields of study – for example, journey mapping is mainly associated with consumer studies and user design, agent-based modelling and network analysis with complexity science and fuzzy cognitive mapping, CLDs and stock-and-flow modelling with system dynamics – they are united explicitly or implicitly by a basis in systems thinking: ‘a set of synergistic analytic skills used to improve the capability of identifying and understanding systems, predicting their behaviours, and devising modifications to them in order to produce desired effects’ [45].

Systems mapping is widely used across many fields as an analytical tool to help improve stakeholders’ capability in understanding complex problems. Systems mapping is an umbrella term for various methods to describe a system’s structure in the form of a diagram. In the field of system dynamics, it typically refers to constructing CLDs, which show causal connections and feedback loops among variables [46]. Systems maps such as CLDs can be generated using qualitative, quantitative or mixed-methods approaches [47–49]. Qualitatively derived systems maps are valued in policy decision-making, particularly when existing data may be incomplete to sustain quantitative simulation models and the issue under consideration needs to take account of the views of multiple with very different experiences of the system [8,10,50,51].

Participatory systems mapping has been described as ‘convening stakeholders in the conceptualisation, analysis and synthesis of knowledge and experience into a useful system diagram or “map”, for the purpose of addressing a messy problem’ [7]. In practical terms, this is typically achieved through stakeholder workshops [7,8,10,52]. Preparation may include identifying stakeholders, gathering evidence, asking guiding questions and building preliminary maps for elaboration. Mapping workshops may involve agreeing the focus issue, defining the boundary of the system, identifying elements of the system and the process of constructing the map itself. Afterwards there may be follow-up activity to refine or digitise the map, interpret it and build verbal narratives to support interpretation. The type of diagram or map produced and the way it is analysed may vary – for example, ‘participatory systems mapping’ has been used to describe the creation of CLDs for the purpose of dynamic simulation [7,10,53], as well as the development of fuzzy cognitive maps for the purpose of network analysis [8,54]. In this paper we use participatory systems mapping to mean involving stakeholders in the process of building and using any kind of systems map.

Antunes et al. [7] outline three kinds of advantage to involving stakeholders in systems mapping: normatively, it can lead to a more democratic process; substantively, it may increase the quality of the map; and pragmatically it can help to build legitimacy and trust in the decision-making process. The participatory process may also yield subjective insights about aspects of the system that different stakeholders consider important, elements that are controllable, areas of vulnerability and/or potential points for intervention [8].

Behavioural systems mapping is an approach to participatory systems mapping designed for the purpose of helping to understand and change human behaviour in complex systems. It involves making explicit the people, behaviours and influences on behaviour within a system and the nature of the relationships among these. As such, it differs from previous approaches by guiding and specifying *what* type of information should be usefully represented in the systems map*. How* the information is represented in terms of the choice of systems mapping method and the visual conventions used can be adapted to reflect the needs of the behavioural problem and system under consideration. In this paper, we describe one of the first uses of behavioural systems mapping, in which the behavioural systems map (BSM) consists of actors (shown using labels), behaviours and influences on behaviour (loosely expressed as variables that may increase or decrease) and causal relationships. Causal relationships in a BSM are depicted using a directed arrow between two variables. In our approach, this represents that the first variable is assumed to effect a mechanistic change in the second variable in a probabilistic manner [55–57]. Constructing a BSM in this way provides a powerful tool for understanding influences on current behaviours, and therefore what needs to be altered at different points in the system in order to bring about change.

To change behaviour within systems, it is also essential to draw upon theory-based behaviour change frameworks to guide intervention design. The BCW is one such framework, developed from a synthesis of 19 previous frameworks. It is based around the capability, opportunity and motivation (COM-B) model, which proposes that three conditions are required for someone to enact a behaviour: capability (attributes of the person, including knowledge and skills), opportunity (attributes of the external environment, including time and resources) and motivation. The COM-B model has previously been used to analyse the factors that affect building repair and maintenance practitioners’ role in retrofit [58,59]. The wider BCW framework is associated with a methodological process for linking an analysis of capability, opportunity and motivation factors to suitable options for behaviour change interventions. This approach has been applied across many disciplinary fields and issues, including: reducing domestic water use [60]; increasing physical activity in school children [61]; reducing sitting time in desk-based office workers [62]; promoting independent living in older adults [63]; supporting parents to reduce provision of unhealthy foods to children [64] and reducing workplace energy use [65]. The first step in applying the BCW is to identify and specify who needs to do what differently (the ‘target behaviour’). This is where behavioural systems mapping may be especially valuable. As representations of the proximal and distal influences on an outcome, BSMs can be used to guide decision-making on which behaviours to target, allowing for assessments of impact and spill-over effects on different options for intervention within the system.

**Figure 1:**
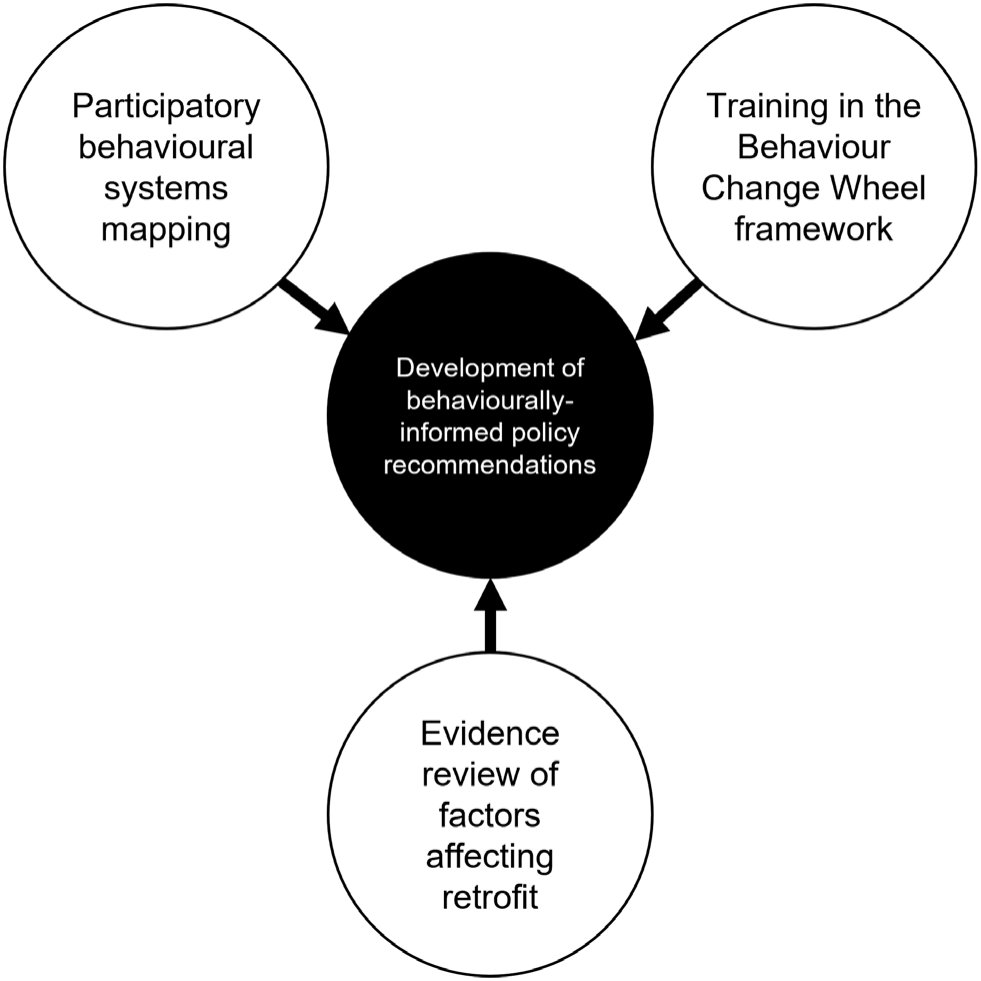
Approach to developing behaviourally-informed policy recommendations.

In previous research and practice there are relatively few examples of bringing together systems mapping and behaviour change frameworks. The aim of this project was to use participatory behavioural system mapping and the BCW framework to synthesise evidence to support the development of behavioural science informed policy recommendations for the retrofit homes in Wales. The project focused on privately owned homes as this sector represents the majority of existing Welsh housing. The main elements of the approach are illustrated in Fig. 1 and described in detail in the following sections. Three questions guided the project: (1) What actors, behaviours and influences on behaviour are involved in the system of decarbonising existing homes in Wales, and what causal pathways connect them? (2) What interventions were identified in the process of drafting policy recommendations? (3) To what extent did draft recommendations address capability, opportunity and motivation influences on behaviours involved in retrofit? Through reporting our steps and outputs in detail it is hoped that others may be able to understand the behavioural systems mapping approach and replicate and adapt the method for their own projects.

## Methods

### Participants

Participants were members of the Advisory Group, which represented 33 organisations (for a full list of member organisations see [21], p. 42). The Advisory Group set up five subgroups, each of which had a nominated chair or co-chair and was responsible for a different part of its remit (technical and infrastructure, governance, community benefits, customer confidence and financial issues). Chairs and co-chairs of each subgroup were invited to participate in three workshops involved in developing policy recommendations, whilst other members of each subgroup were invited to the first workshop only. Due to conflicting commitments, not every member of the Advisory Group was able to participate in every part of the process. Nevertheless, all members were able to review the outputs of workshops or meetings they were unable to attend and input through email or consultations with the subgroup chairs.

## Ethics approval

The methods described here were part of a service delivery project. In accordance with the University College London (UCL) research ethics policy, this work is not classed as research and ethical approval was not sought. A contract between UCL Centre for Behaviour Change and Welsh Government permits the publication of procedures and outputs of participatory workshops. Participants were aware their collective views would be included in publications. No personal data was collected or processed during this project. All work was conducted in accordance with the British Psychological Society Code of Ethics and Conduct.

## Process

The process of drafting of policy recommendations involved a series of reviews, consultations, workshops and data synthesis to reach a final report. Table 1 summarises seven stages in the use of behavioural systems mapping to inform the process, each of which are described in more detail below.

**Table 1.** Stages in the process of developing behaviourally-informed policy recommendations

Stage of the process	Description of activities
Preparatory work	• Review of factors influencing retrofit • Prototype systems map
Workshop 1: Introduction to behaviour change and behavioural systems mapping	• Group behavioural systems mapping • Introduction to the field of behaviour change and the BCW framework
After workshop 1	• Integration of group BSMs • Review of BSMs
Workshop 2: Training in the BCW	• Group review and validation of initial BSMs • Training in the principles of changing behaviour using the BCW framework • Illustration of how to interpret a BSM to identify opportunities for behaviour change interventions
After workshop 2	• Refinement of BSMs^a^ • Drafting of ‘headline’ recommendations^a^
Workshop 3: Development of recommendations	• Identifying interventions needed within sectors^a^
After workshop 3	• Using the COM-B model to refine draft recommendations^a^

^a^Outputs of these activities are presented in the results section.

### Review of factors influencing retrofit

Subgroups of the Advisory Group reviewed literature on factors influencing retrofit across Wales. These were organised into five themes: technical and infrastructure, governance, community benefits, customer confidence, financial issues. On the basis of their reviews, each subgroup was asked to make recommendations for a housing decarbonisation programme.

More details about the content of the subgroup reviews are included in the Advisory Group’s report, *Better Homes, Better Wales, Better World* [21].

### Prototype systems map

A prototype systems map was developed by the authors PC and CJ, which depicted the key groups of actors and behaviours in the Welsh housing system, and the relationships between them at a high level of granularity (Appendix A). This was used as an input to support the participatory mapping process.

### Workshop 1: Introduction to behaviour change and behavioural systems mapping

A one-day workshop was led and facilitated by the authors PC and JH. The objectives of Workshop 1 were:

1. to introduce the field of behaviour change and the BWC framework approach which would be used in the process of developing the recommendations for policy;
2. to share the outputs of the evidence reviews conducted by the Advisory Group subgroups; and
3. to use participatory behavioural systems mapping to synthesise the evidence shared by the subgroups.

The workshop was split into a morning and afternoon session. In the morning session each subgroup shared the main findings of their evidence review, followed by discussion of the implications for decarbonisation policy. In the afternoon, session participants were introduced to the field of behaviour change, the BCW framework and the principles of behavioural systems mapping. They then took part in a group mapping activity to synthesise the information shared in the morning session.

To help guide and focus the behavioural systems mapping activity, an overall ‘target’ behaviour that reflected the Advisory Group’s aims was agreed and specified as ‘a home-owner makes an energy efficient improvement to their home’.

To carry out the behavioural systems mapping activity, the five subgroups were reorganised into six new groups comprising members from each subgroup. Each new group focused on one of two types of privately-owned housing specified in the Advisory Group’s remit (owner-occupiers and private landlords), and one of three sectors identified from the prototype systems map (government, finance, supply chain). Participants chose which group they participated in to reflect their own expertise.

Each group developed a BSM representing the key elements of the system they had been assigned to (e.g., financial sector for owner-occupiers). Based on the evidence shared in the morning session, participants used sticky notes to list actors, behaviours and influences within their assigned sector which could influence decarbonisation of homes. Actors were described as ‘a person or organisation (e.g., builder, domestic energy advisor, mortgage lender, homeowner, government body)’. Behaviours were described as ‘an action that is directly or indirectly observable (e.g., making a decision to install insulation, programming a boiler, attending a course)’. Influences were described as ‘something that affects whether a behaviour is likely to happen (e.g., communication skills, awareness of cost-benefits of a decision, motivation, need for space, market or building regulations)’. Behaviours and influences were subsequently treated as variables in the BSMs, whereas actors were used as labels providing additional information about these variables. Participants were prompted to consider causal relationships between the behaviours and influences they had generated. They were asked to arrange the post-it notes on flipchart paper and draw arrows to indicate the direction of potential causal influence between behaviour and influence variables.

Once participants were satisfied with their maps, the groups rotated to review and give feedback on the maps created by other groups. One person from each group stayed in place to present the map and note the feedback points. Groups incorporated feedback points from other groups into their map.

To reduce complexity and distil the key system features within each sector, the groups were then asked to redraw their map using no more than 10 variables. They were also asked to use arrows to indicate where there may be causal influences between their sector and sectors that other groups had been working on, using the prototype systems map for reference.

At the end of the workshop, photographs were taken of the six group maps and hard copies of the maps were collected.

### Integration of group behavioural systems maps

Two of the authors (PC and JH) integrated the group maps from Workshop 1 into one overall map for owner-occupied homes and one overall map for privately rented homes (social housing was not included in the scope of this project). The overall maps were constructed by entering variables and relationships from the group maps into a diagram in Vensim software (https://vensim.com/). During this process, variable names were refined to make them consistent with the conventions of systems mapping (i.e., could be said to increase/decrease), and any duplicate, unintelligible or variables made redundant by the addition of new variables were discarded. No other changes to the systems map content were made by the researchers.

### Review of behavioural systems maps

The resulting BSMs for owner-occupied and privately rented homes were reviewed by the Chair of the Advisory Group (CJ) and refined based on feedback (e.g., by adding, removing or changing variables and relationships so far as these were identified by the Chair). The maps were distributed for further comment amongst the subgroup chairs and additional feedback was incorporated.

### Workshop 2: Training in the behaviour change wheel

A second one-day workshop was led and facilitated by two authors (PC and JH). The objectives of Workshop 2 were:

1. to train the Advisory Group in the principles of changing behaviour using the BCW framework;
2. to illustrate how to interpret a BSM to identify opportunities for behaviour change interventions; and
3. to review and validate the BSMs for owner-occupiers and private landlords.

Participants received training in the principles of changing behaviour using the BCW framework, which included presentations and group exercises. Then the BSM for private landlords was presented and used to illustrate how the map could be used to identify opportunities for behaviour change interventions. This included highlighting: feedback loops; variables which might influence relatively many others (i.e., an informal appraisal of degree centrality, how many links a node has); and variables which might act as a bridge or gateway influence on others (i.e., an informal appraisal of betweenness centrality, how many times a node lies on the shortest path between others), as well as considering how changes in one part of the system could affect other parts. This analysis was carried out based on visual inspection by the researchers (PC and JH) before the workshop and influence metrics were not formally analysed. After the private landlord map was presented, a validation exercise was conducted in which participants spent time in groups reviewing both BSMs and then gave verbal feedback on them. All feedback points were recorded and used in the final refinement of the maps.

### Refinement of behavioural systems maps

After the second workshop, two of the authors (PC and JH) used the feedback points from the validation exercise to refine the BSMs. The final versions of both maps are presented in the results section.

### Drafting of ‘headline’ recommendations

The Advisory Group continued to meet on a monthly basis following Workshop 2 to further synthesise evidence across subgroups and to begin drafting the recommendations to be put forward to the Welsh Government. Before the next workshop, the Advisory Group chairs drafted a list of 10 ‘headline’ recommendations. These were mapped against the COM-B model by one of the authors (PC) to illustrate how they influenced the capability, opportunity and motivation of owner-occupiers or private landlords to carry out retrofit. The ‘headline’ recommendations and their mapping to the COM-B model are presented in the results section.

### Workshop 3: Development of behaviourally informed policy recommendations

A third one-day workshop was convened and facilitated jointly by the UCL Centre for Behaviour Change and the Advisory Group leads. The objectives of Workshop 3 were:

1. to bring together all learnings so far to develop recommendations to Welsh ministers on the decarbonisation of existing homes;
2. to consider how the drafted recommendations would work together to create the right conditions for the desired objective of decarbonising the Welsh housing sector; and
3. to identify the interventions needed within each sector (government, supply chain and finance) to bring about desired behaviour changes required of owner-occupiers and private landlords.

The 10 ‘headline’ recommendations and their mapping against the COM-B model were presented to participants, who were invited to comment on the ways each recommendation could modify capability, opportunity or motivation.

Participants were then guided through a group exercise to identify the interventions needed within each sector (government, supply chain and finance) to bring about the desired behaviour change goal. This goal was specified as: ‘for owner-occupiers and private landlords to make modifications to improve the energy efficiency of their homes in sufficient numbers as to contribute to a sustainable reduction in the carbon footprint of the existing Welsh housing stock’. Participants were organised into sector specific groups and were given copies of the BSMs and the 10 ‘headline’ recommendations to help them with the exercise.

Each group was asked to identify the three interventions needed to bring about the desired goal in their sector, and how each intervention would change the behaviour of an actor in the decarbonisation system through modifying capability, opportunity or motivation. Each group then shared their work for feedback from other groups. When giving feedback, participants were asked to pay attention to the implications of interventions for their own sector. After receiving feedback, groups revisited their interventions and were asked to consider the implications of them in relation to other sectors (e.g., what needs to change in other sectors to facilitate the identified change?). The final list of identified interventions (draft recommendations) for each sector is presented in the results section. Throughout the activity, participants were asked to think about how each draft recommendation would act to influence behaviour of an actor within the system and to consider discarding or modifying any which could not be identified as having an influence on behaviour of homeowners.

### Using COM-B to refine recommendations

Outputs of Workshop 3 were written up and circulated to the Advisory Group, along with the final versions of the two BSMs for owner-occupied and privately rented homes. The Advisory Group continued to meet to draft the recommendations and sub-recommendations that went into the final report. These were reviewed by one of the authors (PC) to ensure that there were clear causal chains that would influence the target behaviour through reshaping the system to modify capability, opportunity and motivation of key actors within the system. The Advisory Group leads used this review to inform the construction of the final recommendations put forward in the *Better Homes, Better Wales, Better World* report. The final set of recommendations and their mapping to the COM-B model is presented in the results section.

## Results

### What actors, behaviours and influences on behaviour are involved in the system of decarbonising existing homes in Wales and what causal pathways connect them?

**Figure 2:**
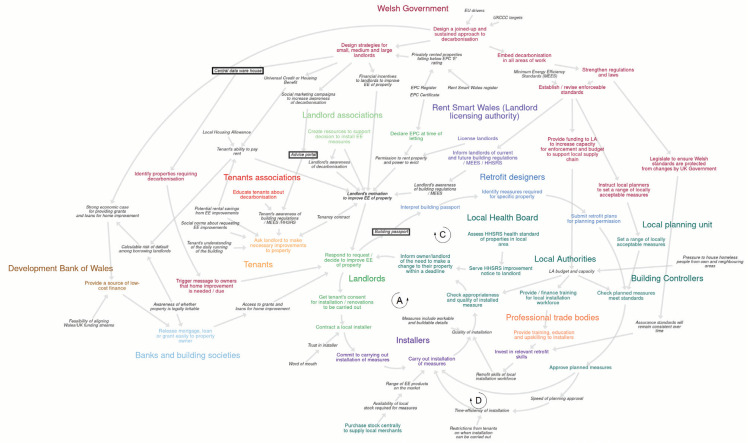
Behavioural systems map for private landlords. *Note.* This figure shows a qualitative BSM for the system of decarbonising homes owned by private landlords. The map represents the system ‘as it could be’, meaning that it reflects the reality of the current system, but it also includes behaviours and influences that could be put in place to support retrofit at scale. Actors are shown in large bold text. Behaviours are shown in medium bold text. Influences on behaviour are shown in small italic text. Colour coding is used to show which actors perform which behaviours. Arrows are used to show positive causal links identified among behaviours and influences on behaviour. Black text boxes are used to show potential new services or resources identified to support decarbonisation. Abbreviations: EE, energy efficiency/energy efficient; EPC, energy performance certificate, HHSRS, housing health and safety rating system; LA, local authority; MEES, minimum energy efficiency standard.

**Figure 3:**
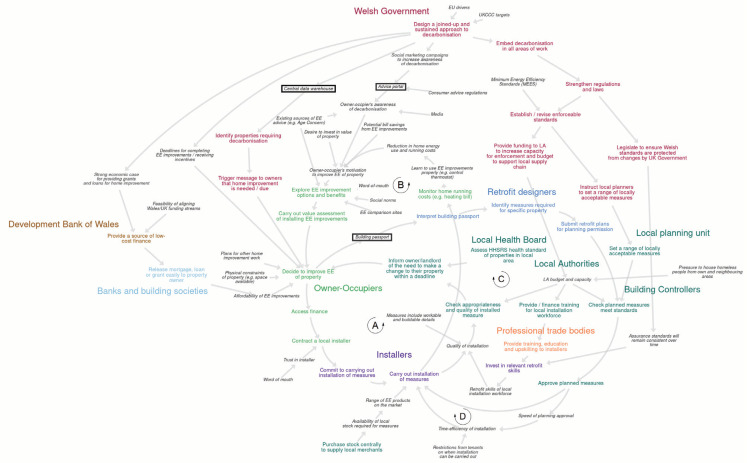
Behavioural systems map for owner-occupiers. *Note.* This figure shows a qualitative BSM for the system of decarbonising homes owned by owner-occupiers. The map represents the system ‘as it could be’, meaning that it reflects the reality of the current system, but it also includes behaviours and influences that could be put in place to support retrofit at scale. Actors are shown in large bold text. Behaviours are shown in medium bold text. Influences on behaviour are shown in small italic text. Colour coding is used to show which actors perform which behaviours. Arrows are used to show positive causal links identified among behaviours and influences on behaviour. Black text boxes are used to show potential new services or resources identified to support decarbonisation. Abbreviations: EE, energy efficiency/energy efficient; HHSRS, housing health and safety rating system; LA, local authority; MEES, minimum energy efficiency standard.

Two BSMs were produced, representing two major housing tenures: private landlords (Fig. 2) and owner-occupiers (Fig. 3). For higher resolution we recommend viewing the maps interactively, online at https://kumu.io/JoHale/walesdecarbonisation.

Across both maps, 26 actors, 43 behaviours and 59 influences on behaviour were identified. Tables B1 and B2 (Appendix B) list all of the actors, behaviours and influences that were identified and included in the map for private landlords and the map for owner-occupiers, respectively.

The two final BSMs are described here, including the main causal pathways shown. Earlier iterations of each map can be found in Appendix B (as images) and at https://osf.io/yekfn/ (as Vensim software files, https://vensim.com/). Each final map represents the system ‘as it could be’, meaning that it reflects the reality of the current system, but it also includes behaviours and influences that could be put in place to support retrofit at scale.

Figure 2 shows the BSMs for private landlords. For a high-resolution interactive version see https://kumu.io/JoHale/walesdecarbonisation#landlords. Actors are shown in large bold text. Behaviours are shown in medium bold text. Influences on behaviour are shown in small italic text. Colour coding is used to show which actors perform which behaviours. Arrows show the causal relationships that were identified between behaviours and influences. Note that this map does not use symbols to indicate polarity, as all causal relationships were ‘positive’ (i.e., both variables change in the same direction).

### Features of the private landlords map

Towards the centre of the map are landlords. In order to retrofit private rented homes, a key behaviour is for landlords to contract a local installer to carry out the work. This is likely to be triggered by a tenant’s request or in response to legislation/regulation, or otherwise deciding to improve the energy efficiency of the property. It may also depend on getting the tenant’s consent for the work to be carried out.

There are three sources of upstream influences on whether landlords will make a decision to improve the energy efficiency of a property. One source of influences comes from tenants, who may influence their landlord by asking them to make necessary improvements to the property. Tenants may be influenced by their understanding of the building, potential savings, social norms about requesting energy efficiency improvements and their awareness of regulations. Tenants could be supported through an advice portal as part of social marketing campaigns and wider strategies from the Welsh Government aimed at small, medium and large landlords.

A second source of influences on the landlord comes from national or devolved government. The landlord’s response/decision will depend on their overall motivation to improve the energy efficiency of the property, which may be influenced by awareness of decarbonisation and buildings regulations such as the minimum energy efficiency standard (MEES), as well as permission to rent the property and the power to evict. Permission to rent is already dependent on declaring the energy performance certificate (EPC) at the time of letting, but could be further used as a lever for the Welsh Government through the licensing of landlords. Another route to support landlords’ motivation could be through the creation of resources by landlord associations.

A third source of influences on landlords comes from the finance sector. It begins with access to grants and loans for home improvement. This may depend on the provision of a source of low-cost finance by the Development Bank of Wales and the easy release of mortgages, loans or grants by banks and buildings societies. In turn, these actions by the finance sector depend on a joined-up and sustained approach to decarbonisation by the Welsh Government.

The fourth source of influence comes from local authorities, which may influence landlords by informing them to make an energy efficiency change to their property within a certain deadline. Informing the landlord could be triggered through the Local Health Board serving a health and safety standards for rented homes (HHSRS) notice or could be triggered by Building Control checks on previously installed energy efficiency measures. This forms a feedback loop labelled ‘A’ in Fig. 2, which can be followed anti-clockwise. Building Control checks would be influenced by local authority budget and capacity, which could be supported through the provision of funding by the Welsh Government. In addition, local authorities would need to set a range of locally acceptable measures, based on enforceable standards established by the national government.

Following on from a landlord’s response/decision to improve the energy efficiency of their property, they may need to get tenants’ permission for works, before contracting a local installer. Whether the installer commits to carrying out the work may be influenced by time-efficiency considerations, affected by the speed of planning approval. Whether they can then carry out the actual installation may depend on the range of energy efficient products on the market and the availability of local stock. Local authorities could purchase stock centrally to help ensure sufficient availability, but they would probably need extra government funding to be able to do so, and they would need to know what products and materials will be in demand. Once carrying out the installation, quality will depend on the retrofit skills of the local workforce. For installers to invest in building retrofit skills, they would need to believe that there will be a steady stream of profitable retrofit work and there would need to be assurance from national government that standards will remain consistent over time. Skills could also be supported by training from professional trade bodies, financed through the local authority.

### Features of the owner-occupiers maps

Figure 3 shows the BSMs for owner-occupiers. For a high-resolution interactive version see https://kumu.io/JoHale/walesdecarbonisation#owner-occupiers. Much of the BSM for owner-occupiers is similar to the map for landlords. Therefore, the parts that differ are described here.

Towards the centre of the map are owner-occupiers. Before owner-occupiers decide to improve the energy-efficiency of their properties, they are likely to explore options and their benefits, and carry out some form of value assessment. Whether they even begin to explore options will depend on their motivation to improve their home’s energy efficiency. Influences on their motivation may include a desire to invest in the property’s value, potential bill savings, awareness of climate change and the importance of decarbonisation and existing sources of advice on energy efficiency. Exploring the options may be influenced by energy efficiency comparison sites, social norms among peers and family and friends and word-of-mouth.

Once an owner-occupier has explored energy efficiency options for their home and carried out a value assessment, whether they decide to actually improve the property may depend on physical constraints such as available space, as well as plans for other home improvement work and deadlines for completing the work (or receiving any government incentives).

Once the owner-occupier has decided to improve the energy efficiency of their property, they may need to access finance before contracting a local installer, who will need to commit to and carry out the installation of the new measures. The owner-occupier may then monitor their home running costs (e.g., their heating bill), helping them to learn to use their energy efficiency improvements properly (e.g., through control of the thermostat). This forms part of the anti-clockwise feedback loop labelled ‘B’ in Fig. 3, whereby seeing a reduction in home energy use and running costs could increase motivation to further improve the property.

### Potential services or resources to support retrofit behaviour change

In both BSMs, three potential services or resources to support decarbonisation were identified, shown as text boxes in the systems map. These are not the only possibilities for influencing retrofit behaviours, but represent potential solutions to ‘gaps’ that were identified during the mapping process. The three potential services or resources would be supported by the design of a joined-up and sustained approach to decarbonisation by the Welsh Government.

First, creation of a central data warehouse could allow the Welsh Government to identify properties requiring retrofit, and could be used to trigger a message to owners that home improvements are needed and/or when they are due. Second, an advice portal for landlords, linked to social marketing campaigns to increase awareness of decarbonisation, could be introduced with the aim of increasing landlord’s motivation to improve the energy efficiency of their property. Finally, Building Renovation Passports (also called Building Renovation Plans [66]) and retrofit designers could be introduced so that when a landlord responds/decides to improve the energy efficiency of their property, a retrofit designer helps to interpret their building passport to identify the measures required for the specific property and submit retrofit plans for planning permission. This could increase the likelihood that the planned measures meet standards and are approved in a timely manner so that an installer can commit to and carry out the work. In this way, retrofit designers are part of two feedback loops connecting the approval of planned measures with committing to the installation (labelled ‘C’ and followed clockwise in Figs 2 and 3), and with carrying out the installation (labelled ‘D’ and followed clockwise in Figs 2 and 3), checking of the appropriateness and quality of the measures by Building Controllers and potentially informing the landlord of the need to make a (further) change to their property.

## What interventions were identified in the process of drafting policy recommendations?

Table 2 lists the key interventions that were identified during Workshop 3 to be needed within government, supply chain and finance sectors to bring about the desired behaviour change of owner-occupiers and private landlords.

**Table 2.** Interventions identified by sector

Sector	Identified interventions
Government	Create a public campaign that is broadly positive in tone, outlining the following features: The climate change emergency is already here, need to safeguard the future of Wales. In order to deal with this emergency, the Welsh Government is going to take several measures that will impact you (or this is happening, regardless of who makes the changes). There are going to be costs involved in making these changes, but also opportunities for the people of Wales. Part of being a responsible government is letting you know what is coming so that you have time to prepare and take advantage of all of the things we are putting in place to help and support you. Some of the benefits need to be articulated in terms of non-climate change and financial – such as increased comfort of the homes. Invest in the Independent Retrofit Advisor Service (i.e., the Welsh Government needs to be seen to lead by example – i.e., retrofitting buildings that they are responsible for, making visible the investment in the form of apprenticeships and finance and funding and making visible trials of retrofit). Build awareness of the importance of carbon-neutral homes into the educational curriculum at all stages, but particularly young people who will enter the housing market in the next 30 years.
Supply chain	Single point of contact (i.e., one phone line/Internet address, needs to be Welsh Government approached, free at point of delivery). Accredited and well-qualified trained and trusted supply chain throughout the system (e.g., surveyors, installers) with accountability built into the system. Holistic, joined-up service. Connecting partners; place-based, where possible.
Finance	Roadmap. Establish a national route map of property-specific, individually, independently assessed journeys to plot how each home is progressed as far as it can. Taxation. Regrade council, land and potentially other tax regimes to reflect energy efficiency and ratchet these more significantly over time. Financial. Enable low-rate finance and funding mechanisms that can be secured with the property and (where appropriate) assigned to third parties for the able to pay, and support for those unable to pay. Enforcement/Regulation. Ensure delivery of energy efficiency measures in practice in homes.

## To what extent did draft recommendations address capability, opportunity and motivation influences on behaviours involved in retrofit?

Table 3 lists the 10 draft ‘headline’ recommendations which were developed after Workshop 2 and coded according to the COM-B model of behaviour. For each recommendation, the final three columns of the table show whether carrying out the recommendation would modify aspects of capability, opportunity and motivation of one or more actors within the system to act in a way that would positively influence the target behaviour–‘a home-owner makes an energy efficient improvement to their home’. Five of the draft recommendations addressed aspects of capability (attributes of the person, including knowledge and skills), opportunity (attributes of the external environment, including time and resources) and motivation. Eight addressed aspects of opportunity. Six addressed aspects of motivation.

**Table 3.** 10 ‘headline’ policy recommendations mapped to the COM-B model

10 ‘headline’ recommendations		Capability	Opportunity	Motivation
1.	Publicly commit now to a 30-year residential decarbonisation programme, with the goal that by 2050 the average energy consumed per Welsh home is less than 6000 kWh per year.	•	•	•
2.	As a first step, find funding solutions to upgrade all homes in fuel poverty by 2030. Privately-owned homes to reach at least EPC C; socially-owned homes to reach at least EPC B.		•	•
3.	In the furtherance of 2, encourage and support social landlords to extend their residential upgrade activities beyond their own portfolios.	•	•	•
4.	Also, in the furtherance of 2, continue WHQS [Welsh Housing Quality Standard] and its £108M pa funding, with a particular requirement to reduce energy demand.		•	
5.	By early 2021 have in place a variety of financial mechanisms to support and encourage the decarbonisation of all homes, across all tenures.		•	•
6.	Encourage and support a quality regime based on PAS 2035 and the resulting British Standard, with only properly trained and accredited individuals allowed to advise on, manage and review the installation of energy conservation measures in homes.	•	•	
7.	Make all publicly-funded loans and grants conditional upon the right measures being installed properly, and all relevant information, including energy consumption data, being made available to the Welsh Government and its agents.	•		•
8.	Encourage and support field trials of ideas (not limited to technical issues) that are considered likely to help decarbonise Welsh homes.	•	•	•
9.	Encourage and support the involvement of: a) Welsh community and voluntary organisations; and b) Welsh SMEs in knowledge sharing and capacity development, so as to maximise jobs, training and supply chains within local communities.	•	•	•
10.	Lobby the UK government hard to support and encourage the further decarbonisation of the electricity and gas supply.		•	

Table 4 lists the 27 final recommendations published by the Advisory Group in their report [21] and shows how each recommendation was coded against the COM-B model. Six recommendations addressed aspects of capability, 24 addressed aspects of opportunity and 12 addressed aspects of motivation.

**Table 4.** Final recommendations mapped to the COM-B model

Final published recommendations		Capability	Opportunity	Motivation
1.1.	Welsh Government to publicly commit now to pursuing a 30-year residential decarbonisation programme.		•	•
1.2.	All politicians at the national and local level should make a clear commitment to supporting the achievement of the targets in Recommendation 2.			•
1.3.	From 2021 all new homes in Wales must be built to be low-carbon, energy and water efficient and climate resilient and independent checks made to ensure higher standards are delivered. This is to prevent the retrofit challenge becoming larger and more expensive to solve.		•	
1.4.	Welsh Government should urgently start developing the recommendations and actions in this report into an ambitious programme of action which is ready for implementation in 2022.		•	
1.5.	Welsh Government should now start urgently – in conjunction with stakeholders and communities in Wales – developing the recommendations from this report into an ambitious programme of action for implementation in 2022.	•	•	•
2.1.	By 2050 the housing stock must be retrofitted to beyond SAP90 (to achieve an EPC Band A rating), recognising that at the moment not all homes will be able to achieve this.		•	
2.2.	Lobby the UK government to support and encourage the further decarbonisation of the energy supply grids. Wales will not achieve the 95% reduction target without it.		•	
2.3.a.	Welsh Government should focus on homes in all tenures, that are currently are below SAP54 (equivalent to EPC Band E, F & G) and specifically ‘off-grid properties’, to help reduce fuel poverty. These homes should be improved to a retrofit target of SAP92 (EPC Band A) rating or beyond wherever possible.		•	
2.3.b.	By 2030 all social homes in Wales should be improved to a retrofit target of SAP92 (to achieve an EPC Band A rating) wherever possible.		•	
3.1.	Fund the creation of and publicly promote a ‘Home LogBook’^a^ for every home to guide energy efficiency decisions and investment. This will include a route map (a set of actions, a timeline and estimated costs) for homes to improve their energy efficiency.	•		•
3.2.	Work with others to create and fund an independent quality regime, reflecting the emerging PAS 2035, that is appropriate for individual homes as well as multi-property projects. Only properly trained and accredited individuals should be allowed to survey, design, specify, project manage, monitor and sign-off the installation of funded energy conservation measures in homes. This should be operational from early 2022.	•	•	•
3.3.	Ensure this regime is appropriate and accessible to SMEs (small and medium-sized enterprises) and sole practitioners in Wales, as well as larger firms. Encourage and support SMEs and sole traders here to develop the skills, capacity and knowledge they need to access the jobs, training, supply chain and other significant opportunities that will arise from this new programme, including innovation in manufacturing, products, services and export opportunities.		•	
3.4.	Encourage and support businesses in Wales to develop opportunities that will result in the best community benefits. To achieve this the procurement arrangements must be based on the Well Being Goals.		•	
3.5.	Ensure as a matter of urgency that the right training schemes, accreditation systems and funding measures etc. are in place.		•	
3.6.	Make all publicly funded loans and grants conditional upon the right measures being properly installed and commissioned.		•	•
3.7.	Encourage and support social landlords to extend their residential upgrade activities beyond their own portfolios to support improvements being undertaken by owner-occupiers and private sector landlords. To do this they should be able to access new funding streams available to homeowners (Recommendation 6) on the owner’s behalf. Social landlords will be subject to the same requirements as any others operating under this regime.		•	•
4.1.	Welsh Government must urgently undertake detailed modelling of the costs associated with the targets set out in Recommendation 2 to inform priority early action including by tenure, archetype and geography.		•	
4.2.	Continue WHQS for social landlords and the £108m pa funding associated with it, on the basis that they are delivering against the stretching targets set out in Recommendation 2.		•	
4.3.	Provide guidance and support to social landlords to enable them to meet the challenging new targets in Recommendation 2.	•	•	
4.4.	Find a financial solution for traditional RSLs who do not currently receive WHQS resources to enable them to meet the stretching targets described in Recommendation 2.		•	
4.5.	Ensure existing public sector funding programmes that support the improvement of homes are aligned with the outcomes and targets recommended in this report.		•	
4.6.	Make resources available to fund the development of Home LogBooks^a^ recommended in Recommendation 3 and the funding of their uptake by private owners.		•	
4.7.	Make the process for homeowners applying for financial support as straightforward as possible, and link it to the need for a Home LogBook^a^ described in Recommendation 3.	•	•	
4.8.	Urgently create financial support mechanisms including low interest loans, to encourage owners (both owner-occupiers and PRS landlords) who wish to improve the energy efficiency of their properties.		•	
5.	All relevant information, including energy consumption data before and after inform measurement of progress, policy development and investment. The data collection process will need to be reviewed in light of the Carbon Delivery Plan.	•		
6.	Establish a fund, to continue at least until 2030, of at least £100m per annum to pay for the development of small and large scale testing of innovative solutions (not limited to technical solutions) that will help to decarbonise Welsh homes. This is particularly vital to test options for homes that are currently without solutions. The Welsh Government’s Innovative Housing Programme (IHP) provides a model for this.		•	
7.	Encourage and support their involvement in the development and delivery of a new programme.		•	•

^a^Home LogBook refers to the same thing as a Building Renovation Passport or Building Renovation Plan.

## Discussion

This project aimed to use methods and frameworks from behavioural science to develop recommendations that placed human behaviour at the centre of national retrofit policy in Wales. Existing approaches drawn from systems research and behaviour change science were adapted and combined to support the drafting of recommendations to government for the content of retrofit housing policy. The project led to the creation of two BSMs representing influences on the retrofit behaviour of private landlords and owner-occupiers. Each map represented the system ‘as it could be’ (i.e., the conditions required to achieve retrofit at scale) and showed causal relationships among the behaviours and actions of people and organisations within national government, local government, the financial sector and the supply chain, as well as private landlords, tenants and owner-occupiers. Three potential interventions included as variables in the maps were: (1) creation of a central data warehouse; (2) an advice portal for landlords; and (3) the use of buildings passports and retrofit designers. Using the maps and their training in the BCW framework, the Advisory Group identified sector-specific interventions that they considered critical to the success of retrofit policy. These interventions contributed to the overall process of developing recommendations. The final recommendations clearly addressed capability, opportunity and/or motivation influences on behaviour, suggesting that the project was successful in helping to develop a policy that is informed by an understanding of human behaviour within a complex sociotechnical system. In September 2019, all the recommendations were accepted on behalf of Welsh Government.

There were at least two sets of benefits from taking a behavioural systems mapping approach to support the Advisory Group’s development of policy recommendations. These related to both the process of taking part in the process used, as well as the outputs it generated. The first set of benefits came from the participatory process of developing the systems maps as a group. Through the discussions involved in creating the maps the Advisory Group members developed a shared representation of their collective knowledge about the issue. Such shared understanding across group members with very different experiences, perceptions and ability to influence an issue is one of the most widely acknowledged benefits of participatory systems mapping, especially in the environmental policy sphere [7,8,10,11,67]. In this project, the workshops were designed to help participants go beyond just assembling their knowledge about different parts of the system like a jigsaw, but to critically interrogate the interrelationships among different subsystems of the wider decarbonisation ecosystem (Workshop 1), thinking about the implications intervening in one part could have on another (Workshop 3). This meant that each subgroup and individual member of the Advisory Group could achieve a richer understanding of the overall system than is represented by the information in the maps alone. Therefore, the process had the benefit of not only generating a shared visual representation, but also developed relationships between group members from different parts of the wider system and supported them to communicate about the issues involved.

Another set of benefits was associated with explicitly specifying actors, behaviours and influences on behaviour when constructing the systems maps. From the outset, we purposively framed the problem in terms of *the human actions that need to change to achieve the desired outcome* (in this case an increase in installing retrofit measures). This kind of framing is fundamental in behaviour change research and practice, and literature on retrofit suggests it is increasingly aligned with how experts view the challenge [4,33,38,40]. There were several advantages to visually mapping how actors, their behaviours and influences on their behaviour are connected within a complex system. Firstly, this provided a coherent ‘common language’ for the Advisory Group to represent their collective knowledge of the retrofit system (Workshop 1). Secondly, it meant that the shared representation (i.e., the whole systems maps) comprised of information that allowed for the use of behavioural science frameworks to be used in the process of designing behaviour change interventions, specifically identifying what behaviour(s) may need to change and selecting appropriate intervention options based on the factors influencing the behaviour(s) (Workshop 2). Thirdly, it allowed the group to anticipate potential intended and unintended consequences of changing a given behaviour within the system, which helped to identify and prioritise the actions to be included in their recommendations (Workshop 3). Overall, this led to policy recommendations that were informed by current understandings of human behaviour within a complex system.

The outputs of this project are specific to the particular context of the Welsh housing system and therefore may have limited applicability to other housing systems, for example, where privately owned homes make up a smaller proportion. Nevertheless, the processes used to generate these outputs may have wider value to other governments considering how to meet the challenge of retrofit to achieve net zero requirements. This is one of the first attempts that we are aware of to frame national scale retrofit as a problem of changing behaviour within in a sociotechnical system. The approach offers a richer understanding of the role and variety of actors in the supply chain, financial sector and national and local government and how they influence each other, addressing an important critique of retrofit policy identified by previous research [25,26,30,42,44]. It is important to note that the researchers closely guided the mapping process so as to direct participants’ attention to actors, behaviours and influences on behaviour, but did not have prior knowledge of the retrofit system which could bias the elements included. The approach has generated insights about causal pathways through which these actors may influence retrofit in the Welsh context, and tangible recommendations for potential interventions which could help to leverage their influence. In addition, the paper adds to existing research using participatory systems mapping to address home energy efficiency, providing practical insights about how to combine systems approaches with frameworks from behavioural science.

This project was the first pilot in the development and use of behavioural systems mapping and therefore had several limitations. The approach intentionally followed conventions of creating CLDs, especially in terms of depicting directed causal relationships between variables. However, several modifications were made to usual processes of constructing and interpreting CLDs which reflected our understanding of the approach at the time. Firstly, the variables in the BSMs were not all specified as values that could increase or decrease (e.g., ‘Minimum Energy Efficiency Standards’). This particularly applies to many of the behaviours (e.g., ‘Access finance’ or ‘Carry out installation of measures’), which are phrased as base verbs implying a one-off action. Maps using this method in future could address this by phrasing behaviour variables as gerunds (e.g., ‘Accessing finance’, ‘Carrying out installation of measures’). Secondly, some of the relationships depicted may not be strictly considered direct cause-effect relationships (e.g., the relationship from ‘Access finance’ to ‘Contract a local installer’) but describe an indirect relationship which is likely to involve mediating variables. Relationships of this nature were included so as to intelligibly represent things which stakeholders considered important influences on behaviour, even if they were not direct immediate causes. Future research or iterations of these maps could represent indirect causal relationships using a different kind of line (e.g., dotted line) or by collecting evidence to develop understanding of the exact causal relationships. Thirdly, a limited number of feedback loops were identified in the BSMs (three in the private landlords map and four in the owner-occupiers map). As feedback loops may drive and explain problematic trends in variables of interest [68,69], this is often a focus of map building workshops [53,70,71]. Whilst our process aimed to identify chains of complex causality it did not explicitly focus on identifying and describing feedback loops. More focus on feedback during the map-building process may have identified more loops which would be helpful when thinking about which elements of the system to amplify or disrupt by policy recommendations.

Compromises are inevitable in any systems mapping project, and the limitations described above reflect a priority to create a map that was useful to the Advisory Group for drafting behaviourally-informed recommendations. Several rounds of review and refinement helped us to be confident in the validity of the maps from their perspective as stakeholders, although it is possible that the maps and recommendations would look different had different stakeholders been involved in each workshop. In order to use BSMs to develop effective behaviour change interventions for retrofit, it would be important to extend and validate this work by systematically eliciting and integrating the perspectives of a wider range of stakeholders. For example, the role of supply chain could particularly benefit from more detailed elaboration about the behaviours of materials and equipment manufacturers, merchants and construction firms [72]. Another important avenue would be to investigate and model different temporal scenarios for the roll-out of retrofit measures, such as measure-by-measure or whole house approaches [28]. The existing BSMs would require considerable improvement and adaptation to act as the basis for quantification and temporal simulation of how the system would change if different scenarios or policy recommendations were implemented. The quality of information in the initial evidence-review process suggested that there was insufficient data to support quantitative modelling at the time this work was completed. This does not mean that systems approaches cannot or should not be applied to answering questions about how to change behaviour. On the contrary, the present work has demonstrated how systems and behavioural approaches can fit together, and has highlighted specific ways that we can improve this fit through methodological improvements.

We welcome collaboration, comment and refinement on our approach. In subsequent work we have refined, developed and applied these methods to a range of other so-called ‘wicked’ problems involving human behaviour. We believe that this method may be a pragmatic approach to answering calls for greater application of systems-based approaches in the field of behaviour change [13–15]. In future work we aim to achieve better ways of representing behaviours and influences on behaviour in CLDs, through collaboration with researchers in system dynamics. We also aim to explore alternative ways of constructing BSMs in contexts where participatory workshops are challenging. These could include practical challenges such as limits on in-person group model building imposed by the global pandemic, [73], and ethical issues associated with bringing together the relevant stakeholders when exploring issues characterised by challenging and abusive behaviour [74]. Furthermore, and in relation to these avenues, we aim to develop more detailed steps for validating BSMs with stakeholders and better ways of transparently documenting their construction and revision. Many of these next steps are already underway through service delivery projects at the UCL Centre for Behaviour Change and through Complex Urban Systems for Sustainability and Health, an international programme of participatory research that brings together behaviour change, system dynamics and other disciplines [3].

## Figures and Tables

**Figure A1:**
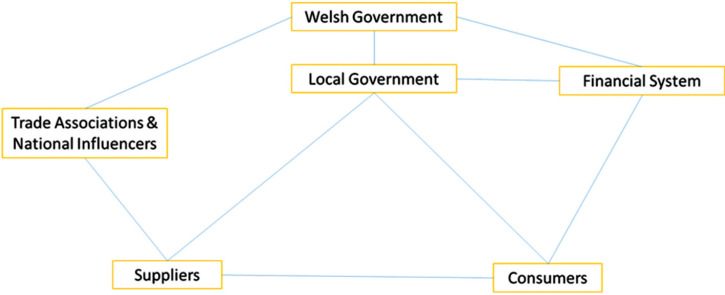
Prototype systems map.

**Figure C1:**
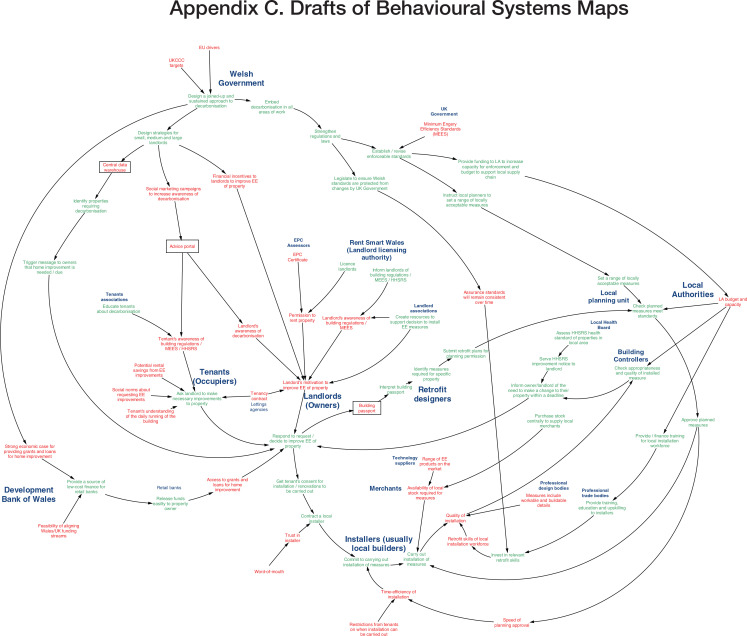
Behavioural systems map for private landlords (draft 1). *Note.* This figure shows a draft BSM for the system of decarbonising homes owned by private landlords. Actors are shown in blue text. Behaviours are shown in green text. Influences on behaviour are shown in red text. Arrows are used to show positive causal links identified among behaviours and influences on behaviour. Black text boxes are used to show potential new services or resources identified to support decarbonisation. Abbreviations: EE, energy efficiency/energy efficient; EPC, energy performance certificate; HHSRS, housing health and safety rating system; LA, local authority; MEES, minimum energy efficiency standard.

**Figure C2:**
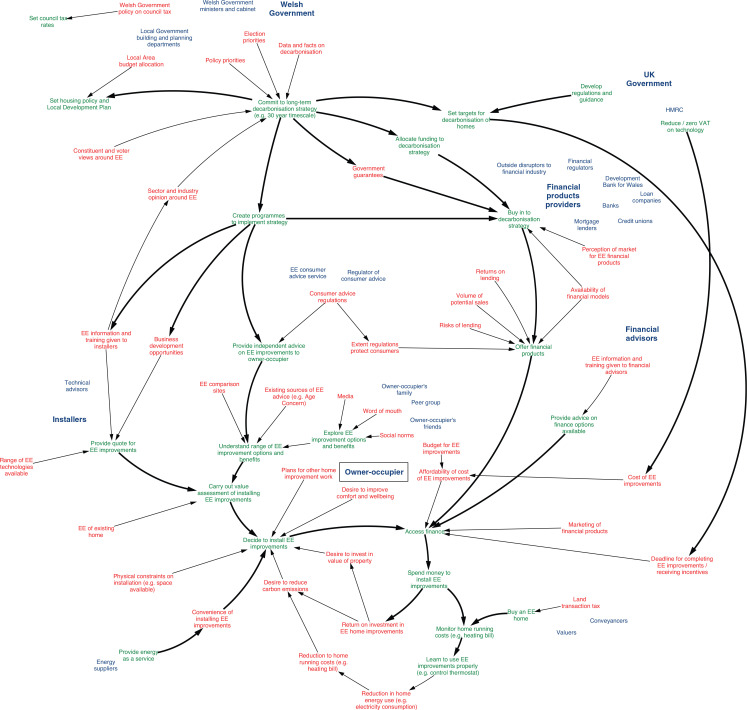
Behavioural systems map for owner-occupiers (draft 1). *Note.* This figure shows a draft BSM for the system of decarbonising homes owned by owner-occupiers. Actors are shown in blue text. Behaviours are shown in green text. Influences on behaviour are shown in red text. Arrows are used to show positive causal links identified among behaviours and influences on behaviour. Abbreviations: EE, energy efficiency/energy efficient; EPC, energy performance certificate; HHSRS, housing health and safety rating system; LA, local authority; MEES, minimum energy efficiency standard.

**Figure C3:**
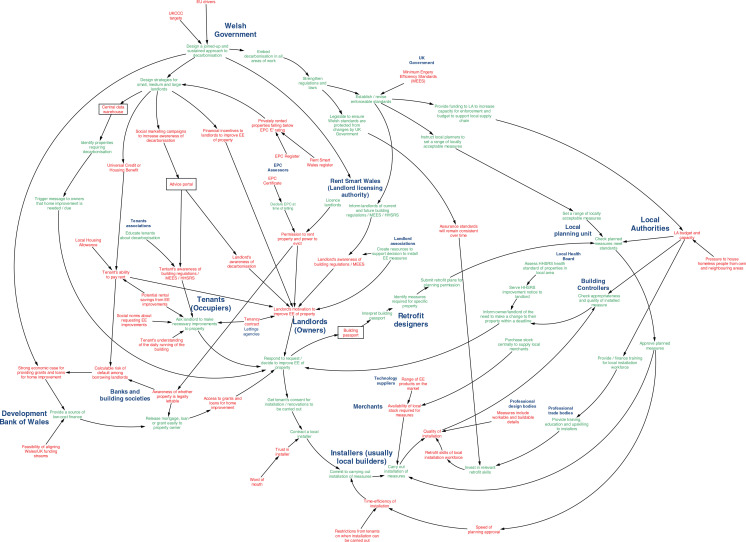
Behavioural systems map for private landlords (draft 2). *Note.* This figure shows a draft BSM for the system of decarbonising homes owned by private landlords. Actors are shown in blue text. Behaviours are shown in green text. Influences on behaviour are shown in red text. Arrows are used to show positive causal links identified among behaviours and influences on behaviour. Black text boxes are used to show potential new services or resources identified to support decarbonisation. Abbreviations: EE, energy efficiency/energy efficient; EPC, energy performance certificate; HHSRS, housing health and safety rating system; LA, local authority; MEES, minimum energy efficiency standard.

## Data Availability

All data generated or analysed during this study are included in this published article (and its supplementary information files). The BSMs shown in Figs 2 and 3 and in Appendix C are available as Vensim software files (https://vensim.com/) at https://osf.io/yekfn/.

## Appendix B. Actors, Behaviours and Influences in the Behavioural Systems Maps

**Table B1.** Actors, behaviours and influences in the BSM for private landlords

Actors linked to behaviours	Behaviours	Influences	Additional actors linked to influences*
Welsh Government	Design a joined-up and sustained approach to decarbonisation	EU drivers UKCCC targets	
	Embed decarbonisation in all areas of work		
	Strengthen regulations and laws		
	Establish/revise enforceable standards	Minimum Energy Efficiency Standards (MEES)	UK Government*
	Provide funding to LA to increase capacity for enforcement and budget to support local supply chain		
	Legislate to ensure Welsh standards are protected from changes by UK Government		
	Instruct local planners to set a range of locally acceptable measures		
	Identify properties requiring decarbonisation	Central data warehouse	
	Design strategies for small, medium and large landlords	Privately rented properties falling below EPC ‘E’ rating Rent Smart Wales register EPC Register	
	Trigger message to owners that home improvement is needed/due		
Local planning unit	Set a range of locally acceptable measures		
	Check planned measures meet standards	LA budget and capacity	
		Pressure to house homeless people from own and neighbouring areas	
	Approve planned measures		
Local Health Board	Assess HHSRS health standard of properties in local area		
	Serve HHSRS improvement notice to landlord		
	Inform owner/landlord of the need to make a change to their property within a deadline		
Building Controllers	Check appropriateness and quality of installed measure	Quality of installation	
		Retrofit skills of local installation workforce	
		Measures include workable and buildable details	Professional design bodies*
Local Authorities	Provide/finance training for local installation workforce		
	Purchase stock centrally to supply local merchants		
Professional trade bodies	Provide training, education and upskilling to installers		
Installers	Invest in relevant retrofit skills	Assurance standards will remain consistent over time	
	Carry out installation of measures	Availability of local stock required for measures	Merchants*
		Range of EE products on the market	Technology suppliers*
	Commit to carrying out installation of measures	Time-efficiency of installation	
		Restrictions from tenants on when installation can be carried out	
		Speed of planning approval	
Retrofit designers	Interpret building passport	Building passport	
	Identify measures required for specific property		
	Submit retrofit plans for planning permission		
Development Bank of Wales	Provide a source of low-cost finance	Strong economic case for providing grants and loans for home improvement	
		Calculable risk of default among borrowing landlords	
		Feasibility of aligning Wales/UK funding streams	
Banks and building societies	Release mortgage, loan or grant easily to property owner	Awareness of whether property is legally lettable	
Landlords	Respond to request/decide to improve EE of property	Landlord’s motivation to improve EE of property	
		Landlord’s awareness of building regulations/MEES	
		Permission to rent property and power to evict	
		Landlord’s awareness of decarbonisation	
		Tenancy contract	Lettings agencies*
		Advice portal	
		Financial incentives to landlords to improve EE of property	
		Social marketing campaigns to increase awareness of decarbonisation	
		Access to grants and loans for home improvement	
	Get tenant’s consent for installation/renovations to be carried out		
	Contract a local installer	Trust in installer	
		Word of mouth	
	Declare EPC at time of letting	EPC Certificate	EPC Assessors*
Tenants	Ask landlord to make necessary improvements to property	Potential rental savings from EE improvements	
		Social norms about requesting EE improvements	
		Tenant’s understanding of the daily running of the building	
		Tenant’s awareness of building regulations/MEES/HHSRS	
		Tenant’s ability to pay rent	
		Local Housing Allowance	
		Universal Credit or Housing Benefit	
Tenants associations	Educate tenants about decarbonisation		
Rent Smart Wales (Landlord licensing authority)	License landlords		
	Inform landlords of current and future building regulations/MEES/HHSRS		
Landlord associations	Create resources to support decision to install EE measures		

Note: *These actors were not linked to any behaviours in the BSM.

**Table B2.** Actors, behaviours and Influences in the BSM for owner-occupiers

Actors linked to behaviours	Behaviours	Influences	Additional actors linked to influences*
Welsh Government	Design a joined-up and sustained approach to decarbonisation	EU drivers	
		UKCCC targets	
	Embed decarbonisation in all areas of work		
	Strengthen regulations and laws		
	Establish/revise enforceable standards	Minimum Energy Efficiency Standards (MEES)	UK Government*
	Provide funding to LA to increase capacity for enforcement and budget to support local supply chain		
	Legislate to ensure Welsh standards are protected from changes by UK Government		
	Instruct local planners to set a range of locally acceptable measures		
	Identify properties requiring decarbonisation	Central data warehouse	
	Trigger message to owners that home improvement is needed / due		
Local planning unit	Set a range of locally acceptable measures		
	Check planned measures meet standards	LA budget and capacity	
		Pressure to house homeless people from own and neighbouring areas	
	Approve planned measures		
Local Health Board	Assess HHSRS health standard of properties in local area		
	Serve HHSRS improvement notice to landlord		
	Inform owner/landlord of the need to make a change to their property within a deadline		
Building Controllers	Check appropriateness and quality of installed measure	Quality of installation	
		Retrofit skills of local installation workforce	
		Measures include workable and buildable details	Professional design bodies*
Local Authorities	Provide/finance training for local installation workforce		
	Purchase stock centrally to supply local merchants		
Professional trade bodies	Provide training, education and upskilling to installers		
Installers	Invest in relevant retrofit skills	Assurance standards will remain consistent over time	
	Carry out installation of measures	Availability of local stock required for measures	Merchants*
		Range of EE products on the market	Technology suppliers*
	Commit to carrying out installation of measures	Time-efficiency of installation	
		Restrictions from owner-occupier on when installation can be carried out	
		Speed of planning approval	
Retrofit designers	Interpret building passport	Building passport	
	Identify measures required for specific property		
	Submit retrofit plans for planning permission		
Development Bank of Wales	Provide a source of low-cost finance	Strong economic case for providing grants and loans for home improvement	
		Feasibility of aligning Wales/UK funding streams	
Banks and building societies	Release mortgage, loan or grant easily to property owner		
Owner-occupiers	Explore EE improvement options and benefits	Owner-occupier’s motivation to improve EE of property	
		Reduction in home energy use and running costs	
		Desire to invest in value of property	
		Potential bill savings from EE improvements	
		Owner-occupier’s awareness of decarbonisation	
		Media	
		Existing sources of EE advice (e.g., age concern)	
		Advice portal	EE Consumer advice service*
		Consumer advice regulations	Regulator of consumer advice*
		Social marketing campaigns to increase awareness of decarbonisation	
		EE comparison sites	
		Social norms	Peer group*
		Word-of-mouth	Family and friends*
	Carry out value assessment of installing EE improvements		
	Decide to improve EE of property	Plans for other home improvement work	
		Deadlines for completing EE improvements/receiving incentives	
		Physical constraints of property (e.g. space available)	
		Affordability of EE improvements	
	Access finance	Marketing of financial products	
	Contract a local installer		
	Monitor home running costs (e.g., heating bill)		
	Learn to use EE improvements properly (e.g., control the thermostat)		

Note: *These actors were not linked to any behaviours in the BSM.
